# Reasons correlated with omission of nursing care*

**DOI:** 10.1590/1980-220X-REEUSP-2022-0171en

**Published:** 2022-11-28

**Authors:** Maria Clara Nascimento Oliveira, Hillda Dandara Carvalho Santos Leite, Vanessa Caminha Aguiar Lopes, João Victor Oliveira Matos Cruz, Claudia Daniella Avelino Vasconcelos, Lídya Tolstenko Nogueira

**Affiliations:** 1 Universidade Federal do Piauí, Centro de Ciências da Saúde, Departamento de Enfermagem, Teresina, PI, Brazil.

**Keywords:** Nursing Care, Patient Safety, Patient Outcome Assessment, Risk Management, Health Evaluation, Atención de Enfermería, Seguridad del Paciente, Evaluación del Resultado de la Atención al Paciente, Gestión de Riesgos, Evaluación en Salud, Cuidado de Enfermagem, Segurança do Paciente, Avaliação de Resultados da Assistência ao Paciente, Gestão de Riscos, Avaliação em Saúde

## Abstract

**Objective:**

To evaluate the reasons correlated with the omission of nursing care in a university hospital.

**Method:**

Analytical cross-sectional design, developed in a university hospital in Northeast Brazil, from January to February 2020. The study population consisted of nurses and nursing technicians who worked in direct patient care. The Brazilian version of the Missed Nursing Care Survey was applied in a convenience sample consisting of 227 participants (79 nurses and 148 nursing technicians). Univariate and bivariate statistics were calculated in the software Statistical Package for Social Science, version 26.0.

**Results:**

The most omitted nursing care was walking three times a day or as prescribed (70.9%). The most prevalent reason was an unexpected increase in the volume and/or severity of patients in the unit (93.0%). Positive, albeit weak, correlations were found between overall care omission, as well as omissions by priority level, and reasons for omission given by nurses and nursing technicians (p < 0.05).

**Conclusion:**

The study showed that the omission of nursing care covered all five dimensions of the instrument, mainly correlated with labor and material resources.

## RESUMO

**Objetivo:**

Avaliar as razões correlacionadas à omissão de cuidados de enfermagem em um hospital universitário.

**Método:**

Delineamento transversal analítico, desenvolvido em um hospital universitário do Nordeste do Brasil, nos meses de janeiro a fevereiro de 2020. A população do estudo compreendeu enfermeiros e técnicos de enfermagem que atuavam na assistência direta ao paciente. Aplicou-se a versão brasileira do *Missed Nursing Care Survey* em uma amostra por conveniência composta por 227 participantes (79 enfermeiros e 148 técnicos de enfermagem). Foram calculadas estatísticas uni e bivariadas no *software Statistical Package for social Science*, versão 26.0.

**Resultados:**

O cuidado de enfermagem mais omitido foi deambulação três vezes por dia ou conforme prescrito (70,9%). A razão mais prevalente foi aumento inesperado do volume e/ou gravidade dos pacientes da unidade (93,0%). Foram verificadas correlações positivas, embora fracas, entre a omissão de cuidados geral, bem como por nível de prioridade, e razões de omissão atribuídas pelos enfermeiros e técnicos de enfermagem (p < 0,05).

**Conclusão:**

O estudo evidenciou que a omissão de cuidados de enfermagem abrangeu todas as cinco dimensões do instrumento correlacionados principalmente aos recursos laborais e materiais.

## RESUMEN

**Objectivo:**

Evaluar los motivos correlacionados con la omisión de cuidados de enfermería en un hospital universitario.

**Método:**

Diseño transversal analítico, desarrollado en un hospital universitario en el Nordeste de Brasil, de enero a febrero de 2020. La población de estudio estuvo compuesta por enfermeros y técnicos de enfermería que actuaban en el cuidado directo del paciente. La versión brasileña de la *Missed Nursing Care Survey en* una muestra por conveniencia compuesta por 227 participantes (79 enfermeros y 148 técnicos de enfermería). Las estadísticas univariadas y bivariadas se calcularon en el *Software Statistical Package for Social Science*, versión 26.0.

**Resultados:**

El cuidado de enfermería más omitido fue caminar tres veces al día o según prescripción (70,9%). El motivo más prevalente fue un aumento inesperado en el volumen y/o gravedad de los pacientes en la unidad (93,0%). Se encontraron correlaciones positivas, aunque débiles, entre la omisión de cuidados generales, así como por nivel de prioridad, y las razones de omisión atribuidas por enfermeros y técnicos de enfermería (p < 0,05).

**Conclusión:**

El estudio mostró que la omisión del cuidado de enfermería abarcó las cinco dimensiones del instrumento, principalmente correlacionadas con los recursos laborales y materiales.

## INTRODUCTION

The global concern with the omission of nursing care derives from the identification of adverse events resulting from delays or non-performance of high, intermediate, and low complexity care in health care, especially in hospitals. This set of facts corroborates the need to direct efforts to understand the reasons why the nursing team omits elements of care in the context of labor and material resources, communication, ethical dimensions, and management style/institutional leadership^(1,2)^.

The nursing team is responsible for patient protection, constantly looking for ways to prevent or minimize harmful actions in the physical, psychological, ethical, and moral spheres. However, this work is challenging, especially in developing countries such as Brazil, and errors in the health care process can and will happen, because to err is human. Therefore, it is important to understand the reasons that favor its occurrence. Thus, ensuring care quality and safety is what drives professionals and leaders in the management of care as the center of the process^(3)^.

Omission of care refers to any care that the patient needs and is not provided or is provided with delay. Thus, when care is performed incorrectly, it will be called acts/errors of omission^(4)^. However, studies have suggested that errors of omission are much more prevalent and harmful^(5,6)^. Omitted care has been identified as a significant predictor for adverse effects related to medication errors, development of pressure injuries and, in more severe cases, death, as well as patient dissatisfaction with the health service, increased hospitalization time, resulting in high expenses for health institutions, and dissatisfaction of nursing professionals^(5,6)^.

Therefore, research has been carried out to understand the reasons given by nursing professionals for the omission of care^(5–7)^. The nursing team plays an important role in providing safe and quality patient care. However, several factors influence this practice, such as infrastructure, service management, staff shortages, work overload, and the insufficient amount of material, a scenario that directly implies an increase in episodes of omission of care, which can lead to adverse events^(7)^.

Considering the importance of investigating the reasons for omission in the context of a university hospital and the positive impact of identifying the omitted care and its associated reasons, allowing the production of essential information to avoid and prevent the continuation of its occurrence, this study aimed to evaluate the reasons correlated with the omission of nursing care in a university hospital. The research question was: “How are the reasons given by nurses and nursing technicians for the omission of nursing care presented?”.

## METHOD

### Design of Study

Analytical cross-sectional design, aiming at measuring the omission of nursing care and verifying the correlated reasons for omission.

### Population and Study Local

The eligible population consisted of 307 nursing professionals (104 nurses and 203 nursing technicians) who worked in the inpatient units and Intensive Care Unit (ICU) of a university hospital in Northeast Brazil. The institution that offers assistance in all specialties of medium and high complexity has 190 beds for hospitalization, 15 beds for ICU and operating rooms, as well as for dental, outpatient, and oral and maxillofacial surgeries. It has been affiliated with the Brazilian Company for Hospital Services (*EBSERH*) since 2013, and operates following the Primary Nursing care model, whose implementation process began in 2015 and was established in 2016^(8)^.

### Selection Criteria

The inclusion criteria were: to exclusively carry out assistance activities in the place and to have at least one year of experience in the institution. Nursing residents and professionals on sick leave during the data collection period were excluded.

### Sample Definition

Participants were selected using the convenience sampling technique and aimed to include professionals who were active and who met the inclusion criteria. Therefore, 227 participants made up the final sample, divided into 79 nurses and 148 nursing technicians. Participants were approached by investigators at the beginning or end of morning, afternoon and evening shifts, who explained the objectives and relevance of the study.

### Data Collection

MISSCARE-BRASIL was developed by Kalisch and Williams in 2009. The Brazilian version of the *Missed Nursing Care Survey* (MISSCARE-Brasil) is an instrument that measures the omission of nursing care and analyzes its causes. It consists of 56 items, distributed in 28 items that question the frequency with which each care is not performed by the nursing team of its unit, measured through a Likert-like scale of five points (1-never performed to 5-always performed), and 28 items investigating the reasons for not performing nursing care in the unit, measured using a Likert-like scale of four points (1-is not a reason to 4-significant reason). In this study, data collection took place from January to February 2020, a period in which the determination of COVID-19 as a pandemic in Brazil was not in force, and the priority classification of omitted care was used, in which care of higher (7 items), intermediate (13 items), and lower priority (8 items) were listed, according to the national reference^(1)^.

The reasons for omission were organized according to the five dimensions accepted in the MISSCARE-Brasil structure: labor resources (8 items), material resources (4 items), communication (10 items), ethical dimension (3 items), and management style/institutional leadership (3 items)^(9)^. Before the analyses, the response codes referring to the items in parts A and B were inverted; thus, higher values corresponded to higher levels of omission and to the most important reasons. The internal consistency values of the five domains of part B of MISSCARE-BRASIL, which corresponded to the validation study of this instrument, resulted in a Cronbach’s alpha greater than 0.70^(7)^.

For the operationalization of the study, a list of nurses and nursing technicians who worked in the inpatient units and in the Intensive Care Units (ICU) was obtained, with no difficulty regarding the acceptance to participate. After receiving explanation about the objectives of the study and signing the consent, the participant received an envelope containing the MISSCARE-Brasil. The mean time for filling out the instrument was 15 minutes. Instructions were given regarding the completion and the participant chose to respond the instruments during the work shift or at home. In these cases, delivery was arranged for the next shift date that the participant had on the work schedule.

### Data Analysis and Treatment

After scores inversion, the answers were dichotomized and, therefore, the alternatives “occasionally omitted”, “rarely performed” and “never performed” implied care omission, and the alternatives “usually performed” and “always performed” represented the care performed. The answers about the reasons were also dichotomized, considering as the reason for omission the options “significant reason” and “moderate reason” and as a nonreason for the omission, “non-significant reason” and “not a reason”. The prevalence of omission of each care was calculated by dividing the number of practices of care omission by the total number of responses that this element obtained, multiplied by 100. The same procedure was used for omission reasons^(4)^.

Data were processed in the software IBM® SPSS® 26.0 and descriptive statistics (mean, standard deviation, minimum, maximum and frequencies) were calculated. In the inferential analysis, the characteristics of the professionals were verified according to the position, using Pearson’s Chi-Square test for qualitative variables and the Student’s t test for quantitative ones. Relationships between omission of care (general and by priority level) and reasons for omission attributed by nurses and nursing technicians were verified using the Pearson’s correlation test. Relationships with p < 0.05 were significant.

### Ethical Aspects

National and international ethical standards for research with human beings were followed. The study was authorized by the university hospital and was approved by the Research Ethics Committee of the Universidade Federal do Piauí, under opinion 3.563.800 in 2019. This research complies with Resolution 466/12 and all participants signed the free and informed consent form, in two copies.

## RESULTS

The sample consisted mainly of females (186; 81.9%) in both professional categories, with no significant difference in age (p = 0.393), whose means (± standard deviation) were 38.5 (± 7 .7) years for nursing technicians and 37.7 (± 5.4) for nurses. The proportion of nursing technicians who had higher education was significant (59; 39.9%) (p < 0.001), with 25 (16.9%) reporting having training in an area other than nursing. Other 44 (29.7%) had a graduate degree or a graduate certificate, in which the majority corresponded to some specialization in the area of nursing (31; 20.9%), and the others in other areas. The frequency of nurses with graduate degree (75; 94.9%) was also significant (p < 0.001).

Most professionals in the nursing staff were assigned to inpatient units (180; 79.3%). Nursing technicians had a significantly longer average time of experience in the sector compared to nurses (4.4 ± 2.0 *versus* 3.7 ± 1.9; p = 0.018). Among the 22 professionals intending to leave the position, 18 (81.8%) were nursing technicians (p = 0.085). There was a significant frequency of nursing technicians among professionals dissatisfied with the position (53; 84.1%; p < 0.001). Among the occupational characteristics, only the sector/unit showed a statistically significant association with the omission of general care (p < 0.001), being pointed out by 96 (94.1%) professionals who worked in the admission units.

The overall prevalence of omission of high-priority nursing care ranged from 56 (24.7%) for airway aspiration to 6 (2.6%) for monitoring capillary blood glucose. Nursing technicians reported a significantly higher frequency of perception regarding the omission of airway aspiration (p < 0.001). The most reported intermediate priority care was ambulation (161; 70.9%), being the most prevalent overall. Higher prevalences were reported by nurses, with the exception of care for skin lesions/wounds, which was more prevalent among nursing technicians (10; 6.8%).

Significant associations were identified for participation in the interdisciplinary team’s discussion about patient care (p = 0.020; 60.8% *versus* 44.6%), patient’s position change every two hours (p = 0.028; 54.4% *versus* 39.2%), fulfillment of requests for administration of prescribed medications within fifteen minutes (p = 0.041; 41.8% *versus* 28.4%), oral hygiene (p = 0.041; 38.0% *versus* 25.0%), patient cleaning immediately after each elimination (p = 0.001; 38.0% *versus* 17.6%), and patient hydration, when appropriate, offering fluids orally or administering through the tube (p = 0.012; 16.5% *versus* 6.1%).

Among the lowest priority care, the prevalence of omission of nursing care ranged from 134 (59.0%), regarding the patient’s seating out of bed, to 24 (10.6%), regarding the use of safety measures for patients at risk of falling. Planning and teaching the patient and/or family for hospital discharge was an element with omission most prevalently reported by nursing technicians (p = 0.041; 33.1% *versus* 20.3%), while complete recording in the patient’s medical record of all necessary data was significantly more reported by nurses (p < 0.001; 27.8% *versus* 9.5%).

As for the reasons for omission, in the labor resources dimension, unexpected increase in the volume and/or severity of patients at the unit (211; 93.0%) and inadequate number of personnel for assistance or administrative tasks (203; 89.4%) were the most prevalent, with agreement between the professional categories. A large number of admissions and discharges (p = 0.029), a high number of nurses with little professional experience (p = 0.047) and a high number of professionals who work sick or with health problems (p = 0.049) were reasons significantly more indicated by the nursing technicians. Nurses, on the other hand, mentioned the professional having more than one job more frequently (p = 0.001).

Regarding material resources, the most prevalent reasons were unavailable materials/equipment (200; 88.1%) or materials that are not functioning properly when necessary (196; 86.3%), with balanced frequencies between groups of professionals. As for communication, unbalanced distribution of patients by professional (163; 71.8%) and non-provision, by other team professionals, of assistance at the time it was necessary (154; 67.8%) were the reasons with the highest frequencies, the former being significantly more perceived by nursing technicians (p = 0.003). In addition, tension/conflict or communication problems with other support departments/sectors (140; 61.7%), within the nursing team (133; 58.6%) and with the medical team (145; 63.9%) presented very similar frequencies among the professionals of the nursing team.

In the ethical dimension, the professional not having an ethical posture and not having commitment and involvement with the work and/or the institution (110; 48.5%) was the most frequent reason, with the others referring to negligence (100; 44.1%) and fear of punishment/dismissal due to job stability (95; 41.9%). As for the management style/institutional leadership, the most prevalent reason was lack of in-service education about the care to be performed (142; 62.6%), followed by nurses’ lack of preparation to lead, supervise and conduct team work (122; 53.7%), being significantly more pointed out by nursing technicians. The relationships observed between the omission of general nursing care and the reasons for omission are shown in Table 1.

**Table 1.** Correlation between general care omission and by level of care priority and the reasons for omission according to the nursing team (n = 227) – Teresina, PI, Brazil, 2020.

Correlation (*r* ^†^)	High	Intermediate	Low	General
**Labor resources**				
Inadequate number of staff	0.070	0.092	**0.153** ^*^	0.121
Patients’ emergency situations	0.096	**0.163** ^*^	0.074	0.128
Unexpected increase in patient volume/severity	0.012	0.010	0.019	0.014
Inadequate number of staff	0.085	0.044	0.111	0.079
Large number of admissions and discharges	0.034	–0.021	0.015	0.005
High number of nurses with little experience	0.074	0.031	0.074	0.065
High number of sick professionals/ with health problems	0.021	–0.004	–0.031	–0.009
The professional has more than one job	0.110	0.115	0.040	0.100
**Material resources**				
Medications not available when needed	**0.144** ^*^	**0.145** ^*^	**0.148** ^*^	**0.163** ^*^
Materials/equipment unavailable when necessary	**0.154** ^*^	0.090	0.101	0.118
Improperly functioning materials/equipment	0.046	–0.082	–0.022	–0.037
Inadequate facilities of the unit/sector	0.046	–0.011	–0.008	0.005
**Communication**				
Unbalanced distribution of patients by professional	0.039	–0.024	–0.022	–0.008
Inappropriate shift handover	0.087	0.093	0.068	0.096
Other team professionals did not provide the assistance	0.070	0.014	0.044	0.040
Team members do not help each other	0.082	–0.014	–0.008	0.008
Tension/conflict/communication problems with other sectors	**0.131** ^*^	0.095	0.071	0.109
Tension/conflict/communication problems within the nursing team	0.079	0.012	0.007	0.026
Tension/conflict/communication problems with medical staff	0.058	0.010	0.014	0.027
Nursing assistant did not communicate that the assistance was not performed	–0.059	–0.081	–0.122	–0.099
Professional responsible for care outside the unit/sector or unavailable	0.090	0.053	0.056	0.067
Lack of standardization for performing procedures/care	0.120	0.006	0.050	0.048
**Ethical dimension**				
Professional without ethical posture/commitment/involvement with work and/or with the institution	0.106	**0.134** ^*^	0.109	**0.140** ^*^
Professional who did not provide care is not afraid of punishment/dismissal due to job stability	0.126	0.025	0.050	0.059
Negligent nursing professional	–0.030	–0.116	–0.112	–0.108
**Management style/institutional leadership**				
Lack of preparation of nurses to lead, supervise, and conduct teamwork	0.013	–0.083	–0.097	–0.073
Lack of in-service education about the care to be performed	0.000	0.031	–0.013	0.010
Lack of motivation for work	0.049	0.116	**0.153** ^*^	0.122

*: P < 0.05; ^†^: Pearson’s Correlation Coefficient.

Positive (direct), but weak correlations were found between the omission of general care, as well as by priority level, and some reasons for omission given by nurses and nursing technicians, whose coefficients ranged from 0.131 to 0.163, in the five dimensions of the MISSCARE-Brazil. There was a weak correlation between “patient emergency situations” and the omission of intermediate-priority care (*r*= 0.163; p = 0.014), in the labor resources dimension.

As for material resources, correlations were found between “medicines not available when needed” and the omission of high-priority (*r*= 0.144; p = 0.031), intermediate (*r*= 0.145; p = 0.029), low (*r*= 0.148; p = 0.026), and general (*r*= 0.163; p = 0.014) care, in addition to “materials or equipment not available when necessary” with high-priority care (*r*= 0.154; p = 0.020). In the communication dimension, a correlation was identified between “tension/conflict and/or communication problems with other sectors” and omission of high-priority care (*r*= 0.131; p = 0.049).

In the ethical dimension, there were correlations between “professional without ethical posture/commitment/involvement with work and/or with the institution” and the omission of intermediate priority care (*r*= 0.134; p = 0.044), as well as general (*r*= 0.140; p = 0.035) care. As for the management style/institutional leadership, a correlation was found between the “lack of motivation for work” and the omission of low-priority care (*r*= 0.153; p = 0.021), as shown in Table 1.

## DISCUSSION

The results obtained evidenced the existence of specific reasons for omission in the context of labor resources, material resources, communication, ethical dimension and management style/institutional leadership correlated with omission at all priority levels of nursing care. At the same time, the direct and weak correlations, combined with the characteristics of the professionals, denote a professional environment with very particular elements that seem to be influenced by the high qualification of professionals and the dynamics of the institution focused on quality management.

In fact, the literature has pointed out that the care omitted may vary according to the professionals’ characteristics, such as educational level, professional category, and experience in the unit^(10)^. In this study, the frequency of professionals with education up to a graduation degree in the position of nursing technicians was very high (69.6%), in addition to having a significant association with dissatisfaction with the position, evidencing one of the differential findings of this study, since the hospital in focus has highly qualified professionals performing mid-level/technical activities, which can be underestimated due to dissatisfaction with the position held, weak commitment to the team and the institution, and increased desire to leave the job.

The association found between a higher prevalence of omission of general care and inpatient units, in this study, can be explained by the wide variety of demands required by each patient, when compared to intensive care units, high physical and mental workload, in addition to the highest general complexity, given that it is a university hospital. The literature shows that intensive care units have teams with longer working time in the sector and stable proportions of nurses for patients who do not need the provision of most low and intermediate priority care^(10)^, which may also have contributed to the result found.

Among the omitted nursing care, intermediate priority prevailed, followed by lower priority and, finally, higher priority. Previous studies indicate that, due to the overload in the work environment, nurses start to prioritize essential care, whose effect would be harmful in the short term^(1,2)^.

Analysis in three countries of nursing care that was omitted, based on Alfaro-Lefevre’s model of care priorities, showed that a lower educational level is associated with a higher frequency of nursing care omission^(10,11)^. In the present study, airway suctioning stood out among high-priority nursing care, in which nursing technicians reported a higher frequency of perception of omission, in view of the technical and care competence presented to identify incidents and question the deficiencies in the nursing service.

The set of intermediate-priority care omitted and significantly perceived by nurses consists of activities that, if occasionally omitted, will not lead to immediate negative outcomes for the patient, such as participation in the interdisciplinary team’s discussion about patient care, meeting of requests for administration of medicines prescribed in fifteen minutes, oral hygiene, cleaning immediately after each elimination, hydration, ambulation and even the patient’s position change every two hours. However, in long-term hospitalizations, the sum of nursing care omissions for the same patient has a strong chance of causing instabilities that can delay the recovery process or even worsen the health condition, increasing the probability of negative outcomes^(8,11)^.

Planning and teaching to the patient and/or family regarding hospital discharge was an element of omission most prevalently reported by nursing technicians in this study. By developing health education with the family and the patient, the team is ensuring continuity of care, adherence, and reduction of the likelihood of readmission to the service^(4)^. In contrast, complete recording in the patient’s medical record of all necessary data was significantly more reported by nurses among the lowest priority care. This negligence comprises a serious problem, since the record protects the professional judicially, regarding the assistance provided, proving care provision^(6)^.

The evaluation of reasons for omission by dimensions of MISSCARE-Brasil showed high prevalence in terms of labor resources, in which the nursing team perceived greater omission due to the large number of admissions and discharges, high number of nurses with little professional experience, high number of professionals who work sick or with health problems, and the professional having more than one job. These data corroborate the results obtained in national and international studies, which showed a significant correlation between the increase in the number of patients per professionals and the increase in the rate of omitted care^(2,4,12)^.

An addition to this research corresponded to the confirmation of the correlation between patients’ emergency situations and the omission of intermediate-level care, especially when considering the context of inpatient units, which are usually not on alert for these cases compared to intensive care units. A Brazilian study highlighted that the greater the degree of dependence of a patient, the greater the number of hours spent on direct and indirect care performed by the nursing team, so the increase in emergency situations will result in more patients demanding a high workload of care under the responsibility of a single professional^(4)^.

The interference of problems related to material resources in the omission of care was evidenced, so that, regardless of the level of care priority, professionals face problems with the unavailability of medicines, materials and equipment during care practice, requiring that related care be performed in the next turn. Materials and equipment shall be in accordance with the specificities of patients treated in a given unit; however, this is still a common problem in different health services^(8,13)^. In addition, the nursing team provides care in a fast-paced and unpredictable environment, which increases the tendency to omit essential care^(14)^.

The correlation found in this study between the omission of care and the existence of tensions, conflicts, or communication problems with other sectors becomes even more important due to its presentation in the set of high-priority care. Health institutions should encourage the interdisciplinary work of multiprofessional teams, aiming at ensuring the quality of care provided and the development of therapeutic approaches directed to comprehensive care^(4)^, providing the reduction of problems in this dimension.

In the ethical dimension, the correlation found between the omission of intermediate-priority care and the existence of professionals without an ethical posture, commitment and involvement with the work and/or the institution is worrying, as professionals with these characteristics may be more prone to absenteeism, overloading the team^(11)^. The imbalances commonly found in health services between the nursing team and organizational factors are recognized as contributing, in a negative way, to the processes of prioritization of care^(15)^; therefore, this professional characteristic can also add negatively to the reasons that lead to the omission of nursing care.

Communication and the ethical dimension comprise independent variables that, when modified, condition an environment that provides professionals’ physical, mental and social well-being^(2)^. Understanding aspects of the work environment is considered an essential element today for health systems, as it expresses the perceptions or feelings of professionals in the face of the institution’s culture and safety climate^(16)^. Understanding this relationship seems to be fundamental to achieving the implementation of effective strategies to favor affective commitment, focus on activities and normative commitment of the nursing team, avoiding a cause and effect network that limits the provision of quality services.

Thus, the existence of a positive environment contributes to the professional practice of nurses and their staff, which is related to lower turnover, absenteeism, and intention to leave the position, as well as greater job satisfaction^(7)^. Feelings of dissatisfaction and the desire to leave the job are found in nursing professionals when they detect the omission of care for their patients, reducing their motivation to work. The services rely on nursing managers identifying and changing the factors influencing the omission of care, ensuring adequate staffing, material resources, and sufficient equipment for care^(13)^. This may be a way to dissolve the correlation between the lack of motivation to work and the omission of low-priority care, in the management style and institutional leadership dimension.

A limitation of the study included convenience sampling, which can reduce the potential for results generalization, although the sample size obtained has shown good responsiveness in statistical inferences. Another limitation can be attributed to the measurement instrument used, which, although validated, presents psychological objects, such as communication, ethical dimension and management style, which can be more reliably evaluated also through specific scales and with evidence of validity.

Correlating the omission of care by priority level with the reasons for omission according to the nursing team allowed us to identify areas that represent potential problems that generate omission in the institution in focus. The results obtained were essential to denote the relevance of investigating the elements of nursing care by priority level, whose relationships can be used by managers and nurse managers to implement specific educational, managerial and service reorganization measures, to avoid omission of nursing care, as well as related negative outcomes, ensuring safe and quality care.

## CONCLUSION

Patients’ urgent situations, unavailability of medicines, materials or equipment when necessary, existence of tension, conflict and communication problems between the nursing team and other sectors, existence of professionals without ethical posture, commitment and involvement with work and/or with the institution, as well as the lack of motivation to work were reasons correlated with the omission of nursing care, distributed among the highest, intermediate or lowest priority levels, as well as when considering the general omission of such care. Positive, albeit weak, relationships encompassed all five dimensions of MISSCARE-Brazil. Notably, the nursing technicians at the university hospital studied have unique occupational characteristics, as they comprise professionals with higher academic qualifications than expected, which should stimulate attention to the influence of this result in future studies.
